# Three weeks of time-restricted eating improves glucose homeostasis in adults with type 2 diabetes but does not improve insulin sensitivity: a randomised crossover trial

**DOI:** 10.1007/s00125-022-05752-z

**Published:** 2022-07-25

**Authors:** Charlotte Andriessen, Ciarán E. Fealy, Anna Veelen, Sten M. M. van Beek, Kay H. M. Roumans, Niels J. Connell, Julian Mevenkamp, Esther Moonen-Kornips, Bas Havekes, Vera B. Schrauwen-Hinderling, Joris Hoeks, Patrick Schrauwen

**Affiliations:** 1 Department of Nutrition and Movement Sciences, NUTRIM School of Nutrition and Translational Research in Metabolism, Maastricht University Medical Center, Maastricht, the Netherlands; 2 Department of Radiology and Nuclear Medicine, NUTRIM School of Nutrition and Translational Research in Metabolism, Maastricht University Medical Center, Maastricht, the Netherlands; 3 Department of Internal Medicine, Division of Endocrinology, NUTRIM School of Nutrition and Translational Research in Metabolism, Maastricht University Medical Center, Maastricht, the Netherlands

**Keywords:** Circadian rhythm, Glucose homeostasis, Hepatic fat, Hepatic glycogen, Insulin sensitivity, Intermittent fasting, Lifestyle intervention, Mitochondrial oxidative capacity, TRE, Type 2 diabetes

## Abstract

**Aims/hypothesis:**

Time-restricted eating (TRE) is suggested to improve metabolic health by limiting food intake to a defined time window, thereby prolonging the overnight fast. This prolonged fast is expected to lead to a more pronounced depletion of hepatic glycogen stores overnight and might improve insulin sensitivity due to an increased need to replenish nutrient storage. Previous studies showed beneficial metabolic effects of 6–8 h TRE regimens in healthy, overweight adults under controlled conditions. However, the effects of TRE on glucose homeostasis in individuals with type 2 diabetes are unclear. Here, we extensively investigated the effects of TRE on hepatic glycogen levels and insulin sensitivity in individuals with type 2 diabetes.

**Methods:**

Fourteen adults with type 2 diabetes (BMI 30.5±4.2 kg/m^2^, HbA_1c_ 46.1±7.2 mmol/mol [6.4±0.7%]) participated in a 3 week TRE (daily food intake within 10 h) vs control (spreading food intake over ≥14 h) regimen in a randomised, crossover trial design. The study was performed at Maastricht University, the Netherlands. Eligibility criteria included diagnosis of type 2 diabetes, intermediate chronotype and absence of medical conditions that could interfere with the study execution and/or outcome. Randomisation was performed by a study-independent investigator, ensuring that an equal amount of participants started with TRE and CON. Due to the nature of the study, neither volunteers nor investigators were blinded to the study interventions. The quality of the data was checked without knowledge on intervention allocation. Hepatic glycogen levels were assessed with ^13^C-MRS and insulin sensitivity was assessed using a hyperinsulinaemic–euglycaemic two-step clamp. Furthermore, glucose homeostasis was assessed with 24 h continuous glucose monitoring devices. Secondary outcomes included 24 h energy expenditure and substrate oxidation, hepatic lipid content and skeletal muscle mitochondrial capacity.

**Results:**

Results are depicted as mean ± SEM. Hepatic glycogen content was similar between TRE and control condition (0.15±0.01 vs 0.15±0.01 AU, *p*=0.88). *M* value was not significantly affected by TRE (19.6±1.8 vs 17.7±1.8 μmol kg^−1^ min^−1^ in TRE vs control, respectively, *p*=0.10). Hepatic and peripheral insulin sensitivity also remained unaffected by TRE (*p*=0.67 and *p*=0.25, respectively). Yet, insulin-induced non-oxidative glucose disposal was increased with TRE (non-oxidative glucose disposal 4.3±1.1 vs 1.5±1.7 μmol kg^−1^ min^−1^, *p*=0.04). TRE increased the time spent in the normoglycaemic range (15.1±0.8 vs 12.2±1.1 h per day, *p*=0.01), and decreased fasting glucose (7.6±0.4 vs 8.6±0.4 mmol/l, *p*=0.03) and 24 h glucose levels (6.8±0.2 vs 7.6±0.3 mmol/l, *p*<0.01). Energy expenditure over 24 h was unaffected; nevertheless, TRE decreased 24 h glucose oxidation (260.2±7.6 vs 277.8±10.7 g/day, *p*=0.04). No adverse events were reported that were related to the interventions.

**Conclusions/interpretation:**

We show that a 10 h TRE regimen is a feasible, safe and effective means to improve 24 h glucose homeostasis in free-living adults with type 2 diabetes. However, these changes were not accompanied by changes in insulin sensitivity or hepatic glycogen.

**Trial registration:**

ClinicalTrials.gov NCT03992248

**Funding:**

ZonMW, 459001013

**Graphical abstract:**

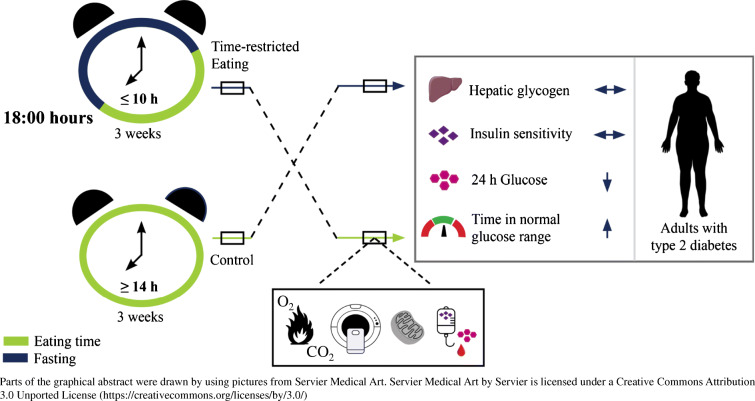

**Supplementary Information:**

The online version of this article (10.1007/s00125-022-05752-z) contains peer-reviewed but unedited supplementary material.

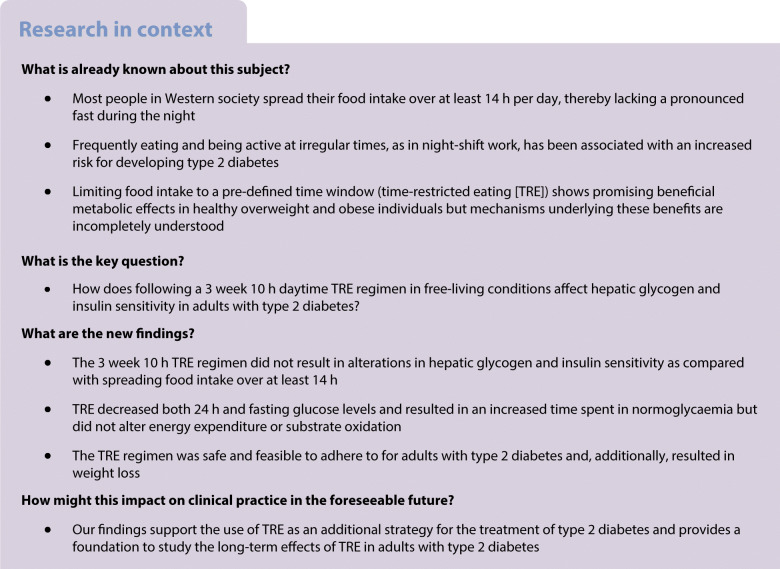



## Introduction

Our modern 24 h society is characterised by ubiquitous food availability, irregular sleep–activity patterns and frequent exposure to artificial light sources. Together, these factors lead to a disrupted day–night rhythm, which contributes to the development of type 2 diabetes [1–3]. In Western society, most people tend to spread their daily food intake over a minimum of 14 h [4], likely resulting in the absence of a true, nocturnal fasted state. Restricting food intake to a pre-defined time window (typically ≤12 h), i.e. time-restricted eating [TRE]), restores the cycle of daytime eating and prolonged fasting during the evening and night. Indeed, several studies demonstrated that TRE has promising metabolic effects in overweight or obese individuals, including increased lipid oxidation [5], decreased plasma glucose levels [6, 7] and improved insulin sensitivity [8]. While promising, the latter studies applied extremely short eating time windows (e.g. 6–8 h) in highly-controlled settings [5–10], thus hampering implementation into daily life. To date, only Parr et al have successfully explored the potential of TRE in adults with type 2 diabetes using a 9 h TRE regimen [11], However, the effects of TRE on metabolic health remained largely unexplored.

Despite the fact that TRE is sometimes accompanied by (unintended) weight loss [4–6, 9, 10, 12, 13], which inherently improves metabolic health, it has also been reported to improve metabolic health in the absence of weight loss [8], indicating that additional mechanisms underlie the effects of TRE. In this context, individuals with impaired metabolic health display aberrations in rhythmicity of metabolic processes such as glucose homeostasis [14, 15], mitochondrial oxidative capacity [16] and whole-body substrate oxidation [16] compared with the rhythms found in healthy, lean individuals [14, 15, 17]. Disruption of circadian rhythmicity is proposed to contribute to the impaired matching of substrate utilisation with substrate availability, which is associated with type 2 diabetes [18]. In turn, we hypothesise that these impairments in metabolic rhythmicity are due to a disturbed eating–fasting cycle. Therefore, restricting food intake to daytime and, consequently, extending the period of fasting, may improve metabolic health. More specifically, hepatic glycogen could play a pivotal role in this process, as it serves as a fuel during the night when glucose levels are low and is replenished during the daytime [19]. A decrease in hepatic glycogen triggers the stimulation of fat oxidation and molecular metabolic adaptations that accommodate substrate availability in the fasted state [20], and the need to replenish these stores may improve insulin sensitivity. Hitherto, it is not known whether TRE could result in a more pronounced depletion in hepatic glycogen levels in type 2 diabetes, leading to an improved insulin sensitivity.

The aim of the current study was to examine the effect of limiting food intake to a feasible 10 h daily time frame for 3 weeks in free-living conditions on hepatic glycogen utilisation and insulin sensitivity in adults with type 2 diabetes.

## Methods

This randomised crossover study was conducted between April 2019 and February 2021, after approval of the Ethics Committee of Maastricht University Medical Center (Maastricht, the Netherlands), and conformed with the Declaration of Helsinki [21]. The trial was registered at ClinicalTrials.gov (registration no. NCT03992248). All volunteers signed an informed consent form prior to participation. The randomisation procedure is described in the electronic supplementary material (ESM) Methods. The study consisted of two 3-week intervention periods separated by a wash-out period of ≥4 weeks. At the end of each intervention period, main outcomes were measured (ESM Fig. 1) at the Metabolic Research Unit of Maastricht University, the Netherlands. Male and female adults with type 2 diabetes, aged between 50 and 75 years and BMI ≥25 kg/m^2^, were eligible for participation. For detailed inclusion and exclusion criteria, see ESM Table 1.

### Intervention

During the TRE intervention, volunteers were instructed to consume their habitual diet within a 10 h window during the daytime, with the last meal completed no later than 18:00 hours. Outside this time window, volunteers were only allowed to drink water, plain tea and black coffee. To increase compliance, volunteers were also allowed to drink zero-energy soft drinks in the evening hours if consumed in moderation. During the control (CON) intervention, volunteers were instructed to spread their habitual diet over at least 14 h per day without additional restraints on the time window of food intake. For both intervention periods, volunteers were instructed to maintain their normal physical activity and sleep patterns and to remain weight stable. Food intake and sleep times were recorded daily using a food and sleep diary. Volunteers based the food intake of their second intervention period on the food and sleep diary filled out during the first period to promote similar dietary quantity and quality in both intervention arms. To optimise compliance, a weekly phone call was scheduled to monitor the volunteers and to provide additional instructions if necessary.

### Procedures

At the start of each intervention period, body weight was determined and a continuous glucose monitoring (CGM) device (Freestyle Libre Pro; Abbott, Chicago, USA) was placed on the back of the upper arm to measured interstitial glucose levels every 15 min. On one occasion, between day 7 and 15 of each intervention, fasted hepatic glycogen was measured in the morning at 07:00 hours using ^13^C-MRS. The day before the MRS measurement, volunteers consumed a standardised meal at home at either 16:40 hours (TRE) or 20:40 hours (CON), ensuring 20 min of meal consumption, so that they were fasted from, respectively, 17:00 hours or 21:00 hours.

On day 19, volunteers arrived at the university at 15:00 hours for measurement of body composition using air displacement plethysmography (BodPod; Cosmed, Rome, Italy), followed by the placement of an i.v. cannula. Afterwards, volunteers entered a respiration chamber for a 36 h measurement of energy expenditure and substrate utilisation using whole-room indirect calorimetry. With TRE, a dinner consisting of 49 per cent of energy (En%) was provided in the respiration chamber at 17:40 hours and volunteers were fasted from 18:00 hours. With CON, a snack of 10 En% was provided at 18:00 hours followed by a 39 En% dinner at 21:40 hours, resulting in a fast from 22:00 hours. Energy content of the meals was based on estimated energy expenditure using the Harris and Benedict equation [22].

On day 20, while in the respiration chamber, a fasted blood sample was obtained at 07:30 hours and 24 h urine was collected for analysis of nitrogen excretion. In both intervention arms, volunteers received standardised meals at fixed times (08:00, 12:00, 15:00 and 18:40 hours) consisting of, respectively, 21 En%, 30 En%, 10 En% and 39 En%. Energy intake was based on sleeping metabolic rate (determined during the night of day 19) multiplied by an activity factor of 1.5. Macronutrient composition of the meals was 56 En% carbohydrates, 30 En% fat and 14 En% protein. Furthermore, volunteers performed low-intensity physical activity at 10:30, 13:00 and 16:00 hours. One bout of activity consisted of 15 min of stepping on an aerobic step and 15 min of standing.

On day 21, after a standardised 11 h fast, volunteers left the respiration chamber at 06:00 hours. Next, a blood sample was taken, followed by measurement of hepatic glycogen and lipid content using ^13^C-MRS and ^1^H-MRS, respectively. Subsequently, a muscle biopsy was obtained to assess ex vivo mitochondrial oxidative capacity, after which a hyperinsulinaemic–euglycaemic two-step clamp was started to measure insulin sensitivity. See ESM Methods for detailed descriptions of measurement methods.

### Biochemical analyses

Blood samples were used for quantification of metabolites and nitrogen was assessed using 24 h urine samples. See ESM Methods for further details regarding the biochemical analyses.

### Data analysis

The statistical packages SPSS Statistics 25 (IBM, New York, USA) and Prism 9 (GraphPad Software, San Diego, USA) were used for statistical analyses. Interventional comparisons are expressed as mean ± SEM. Participant characteristics are expressed as mean ± SD. Differences between CON and TRE were tested using the paired *t* test, unless specified otherwise. A two-sided *p*<0.05 was considered statistically significant. The power calculation, as well as other calculations made using the measured data, are described in the ESM Methods.

## Results

### Participant characteristics

A flowchart of participant enrolment is depicted in ESM Fig. 2. Baseline participant characteristics are presented in Table 1. The median Morningness-Eveningness Questionnaire Self-Assessment (MEQ-SA) score amounted to 59.5 (range 41–72). Only one volunteer was identified as an extreme morning type but was included in the study as the intervention did not interfere with his habitual day–night rhythm.

**Table 1:** Baseline characteristics of participants

Characteristic	Measurement/value
*N*	14
Sex, *n* female/*n* male	7/7
Age, years	67.5±5.2
BMI, kg/m^2^	30.5±3.7
Diabetes medication, *n* yes/*n* no	10/4
Metformin only, *n*	7
Metformin + gliclazide, *n*	3
Fasting plasma glucose, mmol/l	7.9±1.3
HbA_1c_, mmol/mol	46.1±7.2
HbA_1c_, %	6.4±0.7
AST, μkat/l	0.4±0.1
ALT, μkat/l	0.4±0.2
GGT, μkat/l	0.4±0.2
eGFR, ml min^−1^ 1.73 m^−2^	79.9±14.5
MEQ-SA, score	59.1±7.7

Data are shown as mean ± SD, unless stated otherwise

ALT, alanine aminotransferase; AST, aspartate aminotransferase; GGT, γ-glutamyl transferase; MEQ-SA, Morningness-Eveningness Questionnaire Self-Assessment

### Adherence

Volunteers did not indicate any changes in diabetes medication throughout the study. Volunteers recorded their daily food intake and sleep habits for, on average, 17 days during TRE and 18 days during CON. Based on these data, the eating window averaged 9.1±0.2 h in TRE vs 13.4±0.1 h in CON (*p*<0.01). Sleep–wake patterns were similar in both interventions, with a mean sleep duration of 8.1±0.2 h during TRE and 8.0±0.2 h during CON (*p*=0.17). Body weight at the start of each intervention was comparable between TRE and CON (89.1±3.7 vs 89.2±3.8 kg, respectively, *p*=0.62). Although volunteers were instructed to remain weight stable, a small but significant weight loss occurred in response to TRE (−1.0±0.3 kg, *p*<0.01) but not CON (−0.3±0.3 kg, *p*=0.22). The weight loss with TRE was significantly greater than the weight change observed with CON (*p*=0.02). Body composition determined on day 19 was comparable between TRE and CON (TRE vs CON: fat mass 37.4±2.7 vs 37.9±2.9 kg, *p*=0.58; and fat-free mass 50.7±2.6 vs 51.0±2.6 kg, *p*=0.60).

### Hepatic glycogen and lipid content

Approximately half-way through each intervention period, hepatic glycogen levels were assessed in the morning following a 14 h (TRE) and 10 h (CON) night-time fast. Hepatic glycogen did not differ significantly between TRE vs CON (0.16±0.03 vs 0.17±0.02 arbitrary units [AU], respectively, *p*=0.43). At the end of each intervention, hepatic glycogen levels were also assessed after a standardised overnight fast of 11 h for both TRE and CON but did not reveal an altered hepatic glycogen content with TRE compared with CON (0.15±0.01 vs 0.15±0.01 AU, respectively, *p*=0.88). We also assessed hepatic lipid content; neither the amount of lipids nor the composition of the hepatic lipid pool was altered with TRE vs CON (respectively: total lipid content 9.0±2.0 vs 8.6±1.6%, *p*=0.47; polyunsaturated fatty acids 17.0±1.3 vs 16.2±1.2%, *p*=0.41; mono-unsaturated fatty acids 40.6±0.9 vs 42.9±1.4%, *p*=0.19; and saturated fatty acids 42.4±1.2 vs 40.9±1.5%, *p*=0.41).

### Insulin sensitivity and glucose homeostasis

**Fig. 1:**
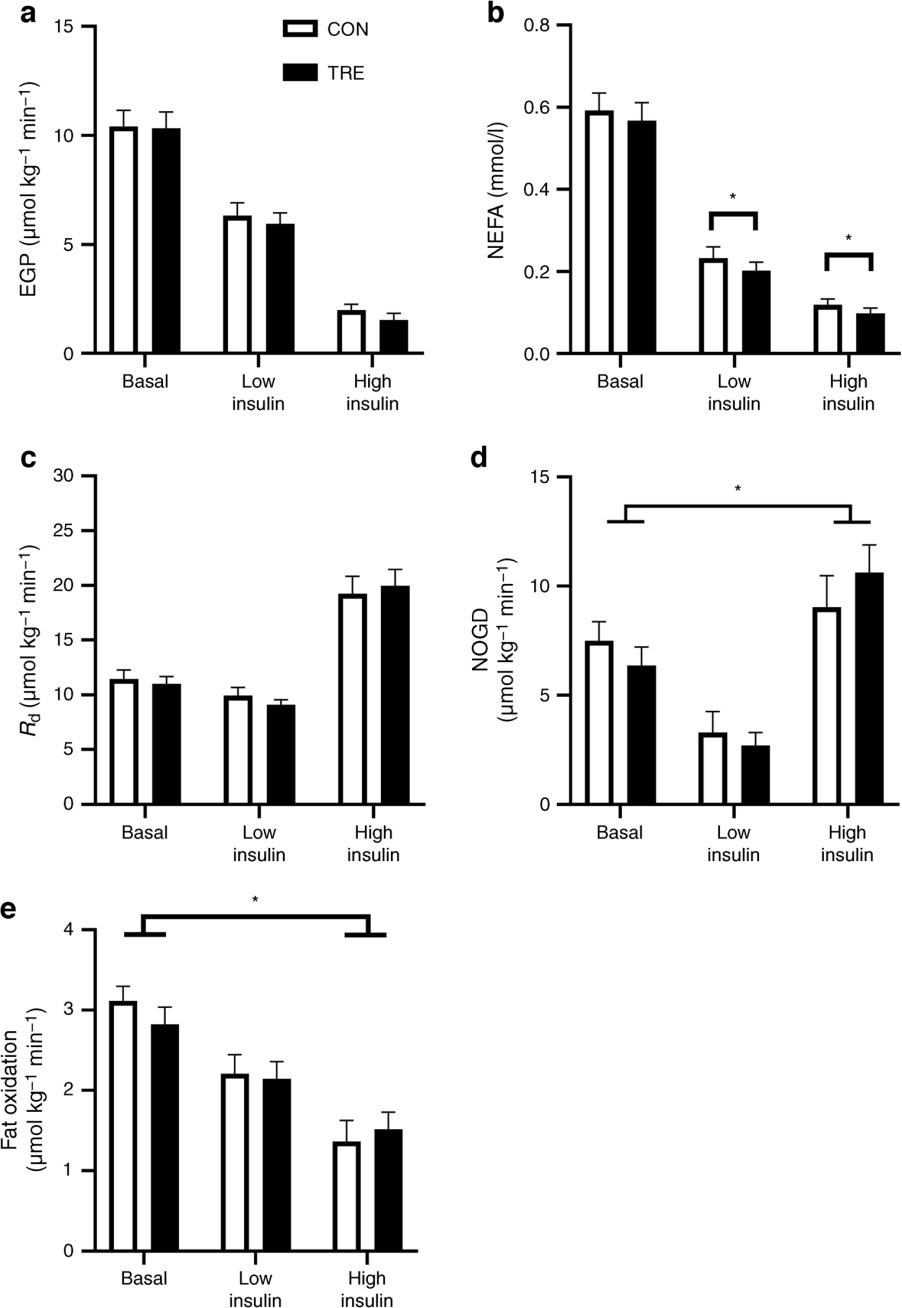
Effect of TRE on EGP (**a**), plasma NEFA (**b**), *R*_d_ (**c**), NOGD (**d**) and fat oxidation (**e**) measured during a hyperinsulinaemic–euglycaemic two-step clamp (*n*=14). *p<0.05 (data were analysed with paired *t* tests). *R*_d_, Rate of disappearance

A hyperinsulinaemic–euglycaemic two-step clamp with a glucose tracer and indirect calorimetry was performed to assess insulin sensitivity. No differences in *M* value were found when comparing TRE and CON (19.6±1.8 vs 17.7±1.8 μmol kg^−1^ min^−1^, respectively, *p*=0.1). Hepatic insulin sensitivity was not affected by TRE, as exemplified by a similar endogenous glucose production (EGP) with TRE and CON in the fasted state and in the low- and high-insulin-stimulated states (*p*=0.83, *p*=0.38 and *p*=0.30, respectively; Fig. 1a). Suppression of EGP was also similar when comparing TRE with CON upon low- and high-insulin infusion (*p*=0.67 and *p*=0.47; Fig. 1a). NEFA suppression upon low insulin exposure was not different between TRE and CON (−365.2±41.6 vs −359.1±43.2 mmol/l, *p*=0.8). However, absolute levels of NEFAs were lower with TRE during the low- and high-insulin phase (*p*=0.02 and *p*=0.04; Fig. 1b), which may hint at an improved adipose tissue insulin sensitivity.

Peripheral insulin-stimulated glucose disposal, reflected by the change in rate of disappearance (*R*_d_) from basal to high insulin, remained unchanged with TRE (*p*=0.25; Fig. 1c). However, we observed a larger insulin-stimulated non-oxidative glucose disposal (NOGD, difference from baseline to high insulin) with TRE than with CON (4.3±1.1 vs 1.5±1.7 μmol kg^−1^ min^−1^, respectively, *p*=0.04; Fig. 1d) reflecting an increased ability to form glycogen. Conversely, insulin-stimulated carbohydrate oxidation from basal to high insulin appeared to be lower with TRE than with CON (4.7±0.9 vs 6.2±0.9 μmol kg^−1^ min^−1^, respectively) but this difference was not statistically significant (*p*=0.07). Consistently, insulin-induced suppression of fat oxidation from basal to high-insulin was lower with TRE than with CON (−1.3±0.3 vs −1.8±0.2 μmol kg^−1^ min^−1^, *p*=0.04; Fig. 1e). Energy expenditure did not differ between TRE and CON during the basal, low-insulin and high-insulin phase of the clamp. These results indicate that while peripheral insulin sensitivity is unchanged with TRE, glucose uptake is more directed towards storage compared with oxidation. Both hepatic and peripheral insulin sensitivity, as well as levels of hepatic glycogen, were additionally analysed in volunteers who only used metformin as diabetes treatment (*n*=7) and this did not alter the outcomes.

**Fig. 2:**
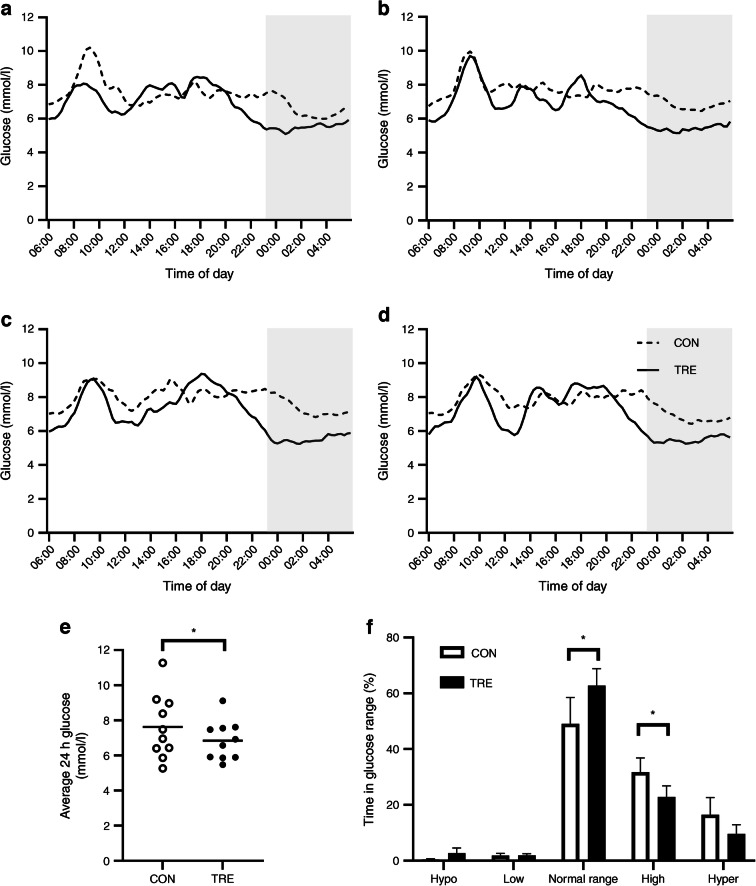
(**a**–**d**) Twenty-four-hour glucose levels on days 15 (**a**), 16 (**b**), 17 (**c**) and 18 (**d**) during TRE or CON (*n*=10). Mean 24 h glucose from day 15 to day 18 (*n*=10) analysed using a paired *t* test (**e**). Time spent in glucose range during days 15–18 (*n*=10) analysed using Wilcoxon tests with Bonferroni correction (**f**) *p<0.05. Hypo, hypoglycaemia defined as glucose levels <4.0 mmol/l; Low, low glucose levels defined as glucose levels 4.0–4.3 mmol/l; Normal range, glucose levels within the normal range defined as 4.4–7.2 mmol/l; High, high glucose levels defined as glucose levels 7.3–9.9 mmol/l; Hyper, hyperglycaemia defined as glucose levels >10 mmol/l

To examine the effect of TRE on glucose homeostasis, CGM data from the last 4 days in the free-living situation (days 15–18) were analysed for both interventions. Four volunteers presented incomplete CGM data due to technical issues, hence statistics were performed on CGM data from ten volunteers. Mean 24 h glucose levels were lower in TRE compared with CON (6.8±0.2 vs 7.6±0.3 mmol/l, *p*<0.01; Fig. 2a–f). Nocturnal glucose levels were consistently lower in TRE vs CON (Fig. 2a–d). Furthermore, volunteers spent more time in the normal glucose range upon TRE compared with CON (15.1±0.8 vs 12.2±1.1 h per day, *p*=0.01; Fig. 2f). Concomitantly, time spent in the high glucose range was less in TRE compared with CON (5.5±0.5 vs 7.5 0.7 h per day, *p*=0.02) whereas no differences between eating regimens were found for time spent in hyperglycaemia (2.3±0.4 vs 3.7±0.8 h per day, *p*=0.24), time spent in the low glucose range (0.5±0.1 vs 0.4±0.1 h per day, *p*=1.00) or time spent in hypoglycaemia (0.7±0.3 vs 0.1±0.0 h per day, *p*=0.48).

Additionally, fasting plasma metabolites were assessed on day 20 and day 21 of each intervention. On day 20, blood samples were taken after a 10 h (CON) or 14 h (TRE) overnight fast. Plasma glucose on day 20 was lower after TRE (7.6±0.4 vs 8.6±0.4 mmol/l, respectively, *p*=0.03) whereas plasma insulin, triglycerides (TG), and NEFA levels were comparable between conditions (Table 2). On day 21, when overnight fasting time was similar for both interventions (11 h), plasma glucose levels remained lower in TRE than in CON (8.0±0.3 vs 8.9±0.5 mmol/l, respectively, *p*=0.04), whereas no differences were detected in plasma insulin, TG and NEFA levels (Table 2).

**Table 2:** Blood plasma biochemistry

Metabolite	CON	TRE	*p* value
Day 20 (*n*=13)			
Triglycerides, mmol/l	2.1±0.3	1.9±0.2	0.30
NEFA, mmol/l	0.529±0.038	0.489±0.035	0.39
Glucose, mmol/l^a^	8.6±0.4	7.6±0.4	0.03
Insulin, pmol/l	111.1±20.8	104.2±13.9	0.27
Day 21 (*n*=14)			
Triglycerides, mmol/l	2.1±0.3	2.2±0.2	0.66
NEFA, mmol/l	0.601±0.070	0.542±0.064	0.30
Glucose, mmol/l^b^	8.9±0.5	8.0±0.3	0.04
Insulin, pmol/l	97.2±13.9	111.1±20.8	0.16

Data are shown as mean ± SEM

^a^Fasted blood values with fasting time 10 h for CON and 14 h for TRE

^b^Fasted blood values with fasting time 11 h for both CON and TRE

### Twenty-four-hour energy and substrate metabolism

**Fig. 3:**
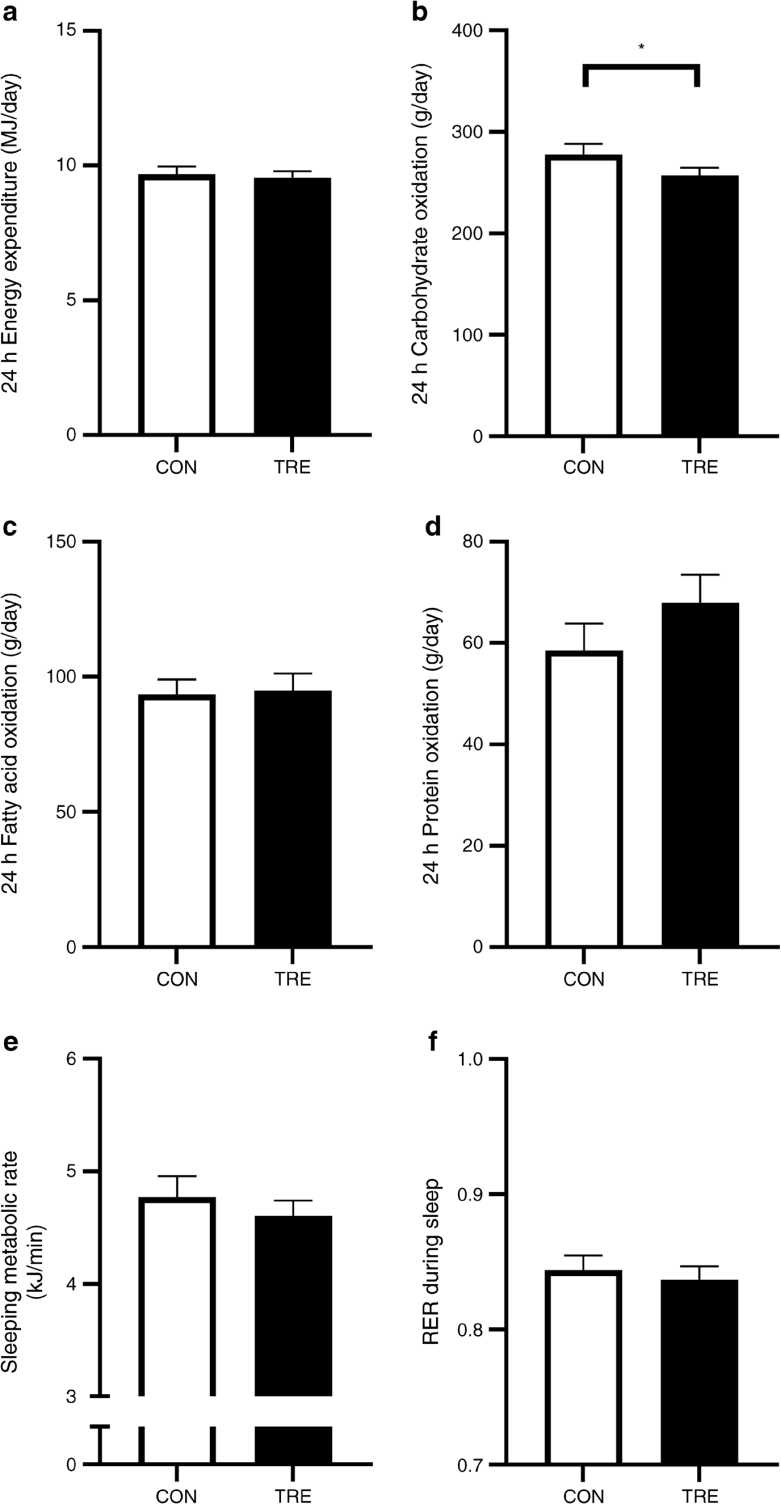
Effect of TRE on 24 h energy expenditure (**a**), substrate oxidation (**b**–**d**), sleeping metabolic rate (**e**) and RER during sleep (**f**), (*n*=13)*.* *p<0.05

On day 19, volunteers resided in a respiration chamber for 36 h for measurement of energy expenditure and substrate oxidation. Twenty-four-hour energy expenditure was similar for TRE and CON (9.57±0.22 vs 9.68±0.29 MJ/day, respectively, *p*=0.22; Fig. 3a), as was the 24 h respiratory exchange ratio (RER) (0.86±0.01 vs 0.86±0.01, respectively, *p*=0.13). Nonetheless, 24 h carbohydrate oxidation was lower in TRE vs CON (260.2±7.6 vs 277.8±10.7 g/day, respectively, *p*=0.04; Fig. 3b), whereas 24 h fat oxidation (91.9±6.6 vs 93.5±5.5 g/day, respectively, *p*=0.72; Fig. 3c) was unaffected. Twenty-four-hour protein oxidation seemed higher upon TRE but the difference did not reach statistical significance (72.8±7.2 vs 58.5±5.4 g/day, respectively, *p*=0.18; Fig. 3d). Sleeping metabolic rate appeared to be lower with TRE compared with CON (4.66±0.14 vs 4.77±0.18 kJ/min, respectively), although this decrease was not statistically significant (*p*=0.05; Fig. 3e). There was no change in carbohydrate or fat oxidation during sleep in response to TRE vs CON (RER 0.84±0.01 vs 0.84±0.01, *p*=0.50; Fig. 3f).

On day 21, muscle biopsies were obtained to assess ex vivo mitochondrial oxidative capacity by means of high-resolution respirometry. In total, paired biopsies from 13 out of 14 volunteers were analysed. Mitochondrial respiration did not differ between TRE and CON (Table 3).

**Table 3:** Mitochondrial oxidative capacity

Respiration state	CON	TRE	*p* value
State 2^a^			
M, pmol mg^−1^ s^−1^	5.0±0.4	5.5±0.6	0.49
MO, pmol mg^−1^ s^−1^	6.6±0.4	6.8±0.5	0.93
MG, pmol mg^−1^ s^−1^	7.1±0.4	6.9±0.4	0.54
State 3^b^			
MO, pmol mg^−1^ s^−1^	28.3±2.0	29.1±1.4	0.91
MG, pmol mg^−1^ s^−1^	31.2±1.9	32.6±1.8	0.82
MOG, pmol mg^−1^ s^−1^	37.0±2.3	37.8±1.9	0.99
MOGS, pmol mg^−1^ s^−1^	55.7±3.3	57.6±2.7	0.80
State U^c^			
MGS, pmol mg^−1^ s^−1^	58.0±3.2	59.9±2.9	0.91
FCCP, pmol mg^−1^ s^−1^	67.8±4.7	66.6±3.5	0.50
State 4o^d^			
Oligomycin, pmol mg^−1^ s^−1^	17.6±1.2	18.1±1.2	0.85

Data presented as mean ± SEM, *n*=13

^a^State 2, respiration in presence of substrates alone

^b^State 3, ADP-stimulated respiration

^c^State U, maximal respiration in response to an uncoupling agent

^d^State 4o, mitochondrial proton leak measured by blocking ATP synthase

FCCP, trifluoro-methoxy carbonyl cyanide-4 phenylhydrazone; G, glutamate; M, malate; O, octanoyl-carnitine; S, succinate

## Discussion

TRE is a novel strategy to improve metabolic health and has been proposed to counteract the detrimental effects of eating throughout the day by limiting food intake to daytime hours. To date, only a few studies have examined the metabolic effects of TRE in adults with type 2 diabetes. Here, we tested whether restricting energy intake to a feasible, 10 h time frame for 3 weeks would lower hepatic glycogen levels and improve insulin sensitivity in overweight/obese adults with type 2 diabetes. Additionally, we explored the effects of TRE on glucose homeostasis, 24 h energy metabolism and mitochondrial function.

We hypothesised that the 10 h TRE regimen, with the latest food intake at 18:00 hours, would result in a more pronounced fasting state, especially during the night. During the night, the liver is crucial to the regulation of blood glucose through the processes of gluconeogenesis and glycogenolysis and it has been shown that these processes are elevated in type 2 diabetes [23, 24]. Therefore, we hypothesised that hepatic glycogen would be lower after TRE and would be associated with an improved insulin sensitivity.

In contrast to our hypothesis, hepatic glycogenolysis appeared to be unaffected by TRE since there was no change in glycogen content after a standardised 11 h fast at the end of the intervention. Neither was there a change after a 14 h (TRE) vs 10 h (CON) overnight fast half-way through the intervention. In addition, EGP suppression during the low-insulin phase of the hyperglycaemic–euglycaemic clamp (reflecting hepatic insulin sensitivity) did not differ between TRE and CON. A limitation of our approach is that we did not measure hepatic glycogen dynamics during the night. Such measurements may be important, as our clamp results showed an increase in NOGD upon high-insulin stimulation with TRE, suggesting an increased glycogen storage. These results could suggest small changes in hepatic glycogen turnover; alternatively, muscle glycogen levels may play a role in explaining our clamp results as the muscle accounts for most of the glycogen synthesis upon high-insulin stimulation in healthy individuals. Interestingly, type 2 diabetes is characterised by an impaired insulin-stimulated glycogen storage [25]. Thus, an improvement in NOGD due to TRE may help to regulate 24 h and postprandial glucose levels. Indeed, 24 h glucose levels were significantly improved after TRE.

We did not observe an effect of TRE on insulin sensitivity. A previous controlled randomised crossover study by Sutton et al did show an improved insulin sensitivity with TRE [8]. Thus, men with prediabetes followed a 5 week 6 h early TRE regimen, whereby the last meal was consumed before 15:00 hours. The differences in results may be explained by the shorter eating window and earlier consumption of the last meal (15:00 vs 18:00 hours), creating a longer period of fasting. Here, we chose a 10 h TRE, which we believe would be feasible to incorporate into the work and family life of most adults with type 2 diabetes; future studies will be needed to reveal whether the duration of the fasting period is indeed crucial in determining positive effects on insulin sensitivity.

Despite the lack of changes in hepatic glycogen and insulin sensitivity, we did find that our 10 h TRE protocol decreased 24 h glucose levels in individuals with type 2 diabetes, primarily driven by decreased nocturnal glucose levels. Notably, TRE also lowered overnight fasting glucose, increased the time spent in the normal glucose range and decreased time spent in the high glucose range, all of which are clinically relevant variables in type 2 diabetes. Importantly, morning fasting glucose levels were consistently lower with TRE than with CON, even when the fasting duration prior to the blood draw was similar between the two interventions. This may indicate lasting changes in nocturnal glucose homeostasis. Additionally, we found that time spent in hypoglycaemia was not significantly increased upon TRE and no serious adverse events were reported resulting from TRE, thereby underscoring that a ~10 h eating window is a safe and effective lifestyle intervention for adults with type 2 diabetes.

Mechanisms underlying the improvement in glucose homeostasis upon TRE remain unclear. Our results show that TRE did not improve peripheral and hepatic insulin sensitivity, skeletal muscle mitochondrial function, energy metabolism or hepatic lipid content, all of which are known to be affected in type 2 diabetes mellitus [25–29]. Under high-insulin conditions during the clamp, we observed a larger reliance on fatty acid oxidation accompanied by higher NEFA levels and lower glucose oxidation. Lower glucose oxidation was also observed when measured over 24 h. Although not statistically significant, the mean of 24 h protein oxidation was higher with TRE, possibly reflecting a more pronounced fasting state and a drive towards a higher rate of amino-acid-driven gluconeogenesis. A previous study by Lundell et al indeed suggested that TRE could affect protein metabolism to cope with the extended period of fasting [30]. However, the exact mechanisms and implications of these effects require further investigation, and it would be interesting to investigate nocturnal glucose metabolism in more detail. The improvement in glucose homeostasis may also partially be explained by the weight loss induced by TRE, which has also been reported previously [4–6, 9, 10, 13, 31]. It should be noted, however, that the body weight loss was rather small (~0.7 kg compared with CON after 3 weeks of intervention) which makes it less likely to completely explain the differences in glucose homeostasis.

A limitation of the current study is the relatively heterogeneous study population consisting of adults with and without use of glucose-lowering medication. Use of medication might have resulted in TRE having less effect, as the medication may be targeting the same metabolic pathways. Only recruiting volunteers not receiving medication would have prevented this issue but would have made the results less applicable to the general type 2 diabetes population. Another limitation of our study is the relatively short duration of 3 weeks. This duration was chosen as the aim of this study was to assess whether TRE would result in metabolic improvements in type 2 diabetes and to explore potential mechanisms underlying these changes. In our experience, human interventions of 3 weeks are able to affect the outcome variables investigated in our study. Since our TRE protocol was feasible and safe, and resulted in improved 24 h glucose levels, it would be interesting to examine the impact of 10 h TRE on glucose homeostasis and insulin sensitivity in type 2 diabetes in the long term to address the clinical relevance of TRE.

In conclusion, we show that a daytime 10 h TRE regimen for 3 weeks decreases glucose levels and prolongs the time spent in normoglycaemia in adults with type 2 diabetes as compared with spreading daily food intake over at least 14 h. These improvements were not mediated by changes in hepatic glycogen, insulin sensitivity, mitochondrial function or 24 h substrate oxidation. These data highlight the potential benefits of TRE in type 2 diabetes.

## Supplementary information

ESM (PDF 809 kb)

## Data Availability

The datasets that were obtained in this study can be made available by the corresponding author upon reasonable request.

## Abbreviations

AU: Arbitrary units
CGM: Continuous glucose monitoring
CON: Control
EGP: Endogenous glucose production
En%: Per cent of energy
NOGD: Non-oxidative glucose disposal
RER: Respiratory exchange ratio
SMR: Sleeping metabolic rate
TG: Triglycerides
TRE: Time-restricted eating
