# Supplementary material for: Three weeks of time-restricted eating improves glucose homeostasis in adults with type 2 diabetes but does not improve insulin sensitivity: a randomised crossover trial
Source: Diabetologia. 2022 Jul 25;65(10):1710–20. doi: 10.1007/s00125-022-05752-z (PMC9477920; doi:10.1007/s00125-022-05752-z)
Supplement: Supplementary file 1 — (PDF 809 kb) [file 125_2022_5752_MOESM1_ESM.pdf]

**Electronic supplementary material**

# METHODS

## Research Design and Methods

Prior to the study, a randomisation schedule was constructed by a study-independent researcher using the website randomizer.org. Intervention sequences (TRE:CON or CON:TRE) were constructed using blocks of equal size which ensured that an equal number of patients started with CON as with TRE. Due to the nature of the study, neither coordinating investigator nor patients were blinded to the intervention. When volunteers were found eligible to participate, study enrolment and allocation to either TRE and CON (based on the randomisation schedule) was performed by the coordinating investigator.

## Procedures

A 3.0 T clinical MRI scanner (Achieva Philips Healthcare, Best, the Netherlands) was used to perform the measurements of hepatic glycogen and hepatic lipid content. Glycogen was measured using $^{13}$C-MRS with a dedicated $^{13}$C / $^{1}$H coil and the volunteer lying in prone position. Power settings were calibrated to achieve a 90-degree pulse in the liver (at 8 cm from the coil) and spectra were acquired without 1H-decoupling. The area under the curve of the glycogen doublet at 100.5 ppm was determined using MATLAB and phantom-based sensitivity maps and MRI image segmentation were used to correct for coil sensitivity in the liver area. For hepatic lipid content, volunteers were positioned in the supine position and a volume of interest was selected within the right lobe of the liver to acquire $^{1}$H-MRS spectra using STEAM (TE: 20 ms, TR: 4500ms, number of averages: 128) [1]. Volunteers were asked to breathe in a rhythm to prevent motion artefacts. The water signal was suppressed by VAPOR water suppression. A spectrum without water suppression was also acquired to quantify the water signal and the ratio of lipid over the sum of lipid and water was determined. From this, absolute values of fat percentage were deduced [2] and hepatic lipid content is given as weight/weight percentage. From the lipid spectrum, the relative contribution of saturated, monounsaturated and polyunsaturated fatty acids were determined according to Roumans et al [1].

To measure mitochondrial oxidative capacity, permeabilized muscle fibres were prepared freshly directly after the muscle biopsy as described previously [4]. Subsequently, the permeabilized muscle fibres (~2.5 mg wet weight) were analysed for mitochondrial function using an oxygraph (OROBOROS Instruments, Innsbruck, Austria). To prevent oxygen limitation, the respiration chambers were hyper-oxygenated up to ~400 µmol $L^{-1}$ $O_2$. Subsequently, two different multi-substrate/inhibition protocols were used in which substrates (malate, octanoyl-carnitine, glutamate, succinate) and other compounds (ADP, oligomycin, FCCP) were added consecutively at saturating concentrations to characterize mitochondrial capacity, as described previously [5]. Measurements were performed in quadruplicate and cytochrome c was added upon maximal coupled respiration (state 3) to assess mitochondrial membrane integrity. If oxygen consumption increased >15% after cytochrome c addition, that particular measurement was excluded from analysis.

The hyperinsulinemic-euglycemic two-step clamp was performed to measure insulin sensitivity, as described previously [3]. Briefly, the clamp started with 120 minutes of primed-continuous infusion of D-[6,6-$^{2}H_2$] glucose to determine baseline endogenous glucose production (EGP), glucose appearance (Ra) and glucose disposal (Rd). Afterwards, insulin

was infused at 10 mU · $m^2$ · $min^{-1}$ to assess hepatic insulin sensitivity reflected by suppression of EGP. After 3 h, insulin infusion was increased to 40 mU · $m^2$ · $min^{-1}$ to measure muscle insulin sensitivity. Arterialized blood was drawn every 5-10 minutes to assess glucose levels and glucose (20%) was co-infused to maintain glucose levels at ~5 mmol/l. Energy expenditure and substrate utilization was measured during the last 30 min of every steady-state period (basal, low insulin and high insulin) using indirect calorimetry (Omnical; Maastricht Instruments, Maastricht, the Netherlands).

**Biochemical analyses**

The ABX Pentra C400 (Horiba, Montpellier, France) was used to enzymatically quantify triglycerides (Sigma, St Louis, USA), free fatty acids (FFAs) (Wako, Neuss, Germany) and glucose concentrations (Horiba, Montpellier, France) in EDTA plasma. Insulin levels were determined using enzyme-linked immunoassay in EDTA plasma (Crystal Chem Inc, Illinois, USA). Nitrogen was assessed in 24-hour urine samples using the Vario Max (Elementar Analysensysteme GmbH, Langenselbold, Germany). Samples from volunteers were analysed in the same run for both interventions.

**Data analysis**

For determination of glucose homeostasis, continuous glucose monitor data was obtained in the free-living situation of both TRE and CON. Data from the last 4 days (day 15 – 18) was combined to account for day-to-day specific effects on glucose excursions. This continuous data was divided into categories defined by the American Diabetes Association [6, 7]: hypoglycaemia < 4.0 mmol/l, low blood glucose 4.0 – 4.3 mmol/l, normal range 4.4 – 7.2 mmol/l, high blood glucose 7.3 – 9.9 mmol/l, hyperglycaemia > 10.0 mmol/l. Results were reported as percentage of time spent in the respective categories and differences between TRE and CON were tested using multiple Wilcoxon signed rank tests with a Bonferroni correction.

Sleeping metabolic rate was defined as the lowest 3 h of nocturnal energy expenditure during the first night in the respiration chamber and calculated with the Weir equation [8]. Twenty-four-hour energy expenditure and -substrate utilization were calculated using the equation from Brouwer et al. [9] with data obtained from the last 24 h of the respiration chamber measurement, including 24-hour urine collection to determine protein oxidation. From the indirect calorimetry data collected during the clamp, carbohydrate- and fat oxidation were calculated using the Brouwer equation [9] with protein oxidation being estimated as 12.4% of energy expenditure. In addition, Steele's singe pool non-steady state equations were used to calculate Ra and Rd [10].

Sample size was determined based on the variability in glycogen content measured in the fasted state with $^{13}$C-MRS, which amounted 7 – 11% in previous studies [11, 12]. For a more conservative estimation of the standard deviation, 11% was used in our calculation. The following equation was used for sample size calculation: $N = \sigma^2/\Delta\mu^2 * (Z_{0.8} + Z_{0.975})^2$ with $Z_{0.8} = 0.842$, $Z_{0.975} = 1.960$, $\sigma^2 = 11\%$, and $\Delta\mu^2 = 10\%$. Filling out this equation indicated that we needed to included 10 volunteers in our study. However, since the variation in hepatic glycogen in response to a time restricted eating regime was not investigated at the time of the calculation and might be greater, we decided to include 14 volunteers in our final data analyses.

## TABLES

*ESM Table 1: Inclusion criteria of the trial*

| **Inclusion criteria** | |
|---|---|
| **1** | Patients are able to provide signed and dated written informed consent prior to any study specific procedures |
| **2** | Caucasian |
| **3** | Non-insulin treated type 2 diabetes |
| **4** | Women are post-menopausal (defined as at least 1 year cessation of menses) |
| **5** | Age: 50 – 75 years |
| **6** | BMI ≥ 25 kg/m$^2$ |
| **7** | Regular sleeping time (normally 7 – 9h daily) |
| **8** | Habitual sleeping time 11 PM ± 2 h |
| | |
| **Exclusion criteria** | |
| **1** | Not being able to adhere to a restricted eating schedule |
| **2** | Uncontrolled hypertension |
| **3** | Active cardiovascular disease |
| **4** | Insulin therapy |
| **5** | Use of sodium-glucose costransporter-2 inhibitors |
| **6** | Engagement in programmed exercise for >3h per week |
| **7** | Extreme early bird or extreme night person (score ≤30 or ≥70 on morningness-eveningness questionnaire – self assessment questionnaire) |
| **8** | Heavily varying sleep-wake rhythm |
| **9** | Shiftwork during last 3 months |
| **10** | Smoking |
| **11** | Contra-indication to Magnetic Resonance Imaging (MRI) scanning |
| **12** | Blood donation during intervention or less than three months before the start of intervention |
| **13** | Not willing to be informed about unexpected medical findings during the screening/study, or not willing to have the attending general practitioner informed about study (findings) |
| **14** | Unstable body weight (weight gain or loss > 3 kg during 3 months prior to study onset) |
| **15** | Significant food allergies/intolerance (seriously hampering study meals) |
| **16** | Participation in another biomedical study within 1 month before the first study visit, which would possibly hamper our study results |
| **17** | Another medical condition that will preclude the safe performance of the measurements as judged by the medical doctor |

**FIGURES**

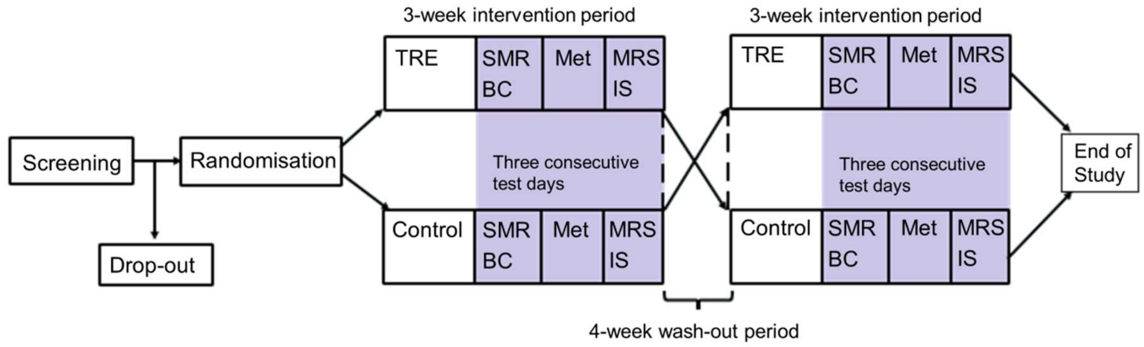


**ESM Figure 1** – Overview of the study design of the time restricted eating study. TRE: time restricted eating, SMR: sleeping metabolic rate, BC: body composition, Met: 24-hour energy metabolism, MRS: magnetic resonance spectroscopy, IS: insulin sensitivity

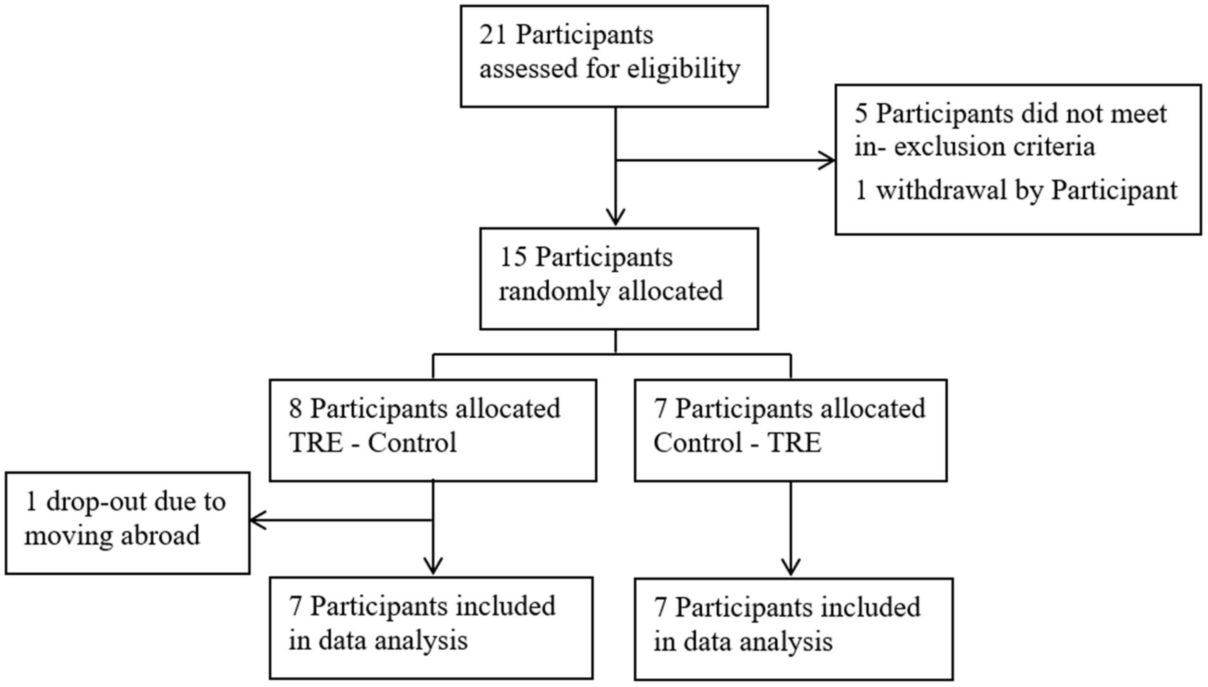


**ESM Figure 2** – Flowchart of the time restricted eating study

## REFERENCES

[1] Roumans KH, Lindeboom L, Veeraiah P, et al. (2020) Hepatic saturated fatty acid fraction is associated with de novo lipogenesis and hepatic insulin resistance. Nature communications 11(1): 1-11

[2] Szczepaniak LS, Babcock EE, Schick F, et al. (1999) Measurement of intracellular triglyceride stores by H spectroscopy: validation in vivo. American Journal of Physiology-Endocrinology And Metabolism 276(5): E977-E989

[3] Van De Weijer T, Phielix E, Bilet L, et al. (2015) Evidence for a direct effect of the NAD+ precursor acipimox on muscle mitochondrial function in humans. Diabetes 64(4): 1193-1201

[4] Phielix E, Schrauwen-Hinderling VB, Mensink M, et al. (2008) Lower intrinsic ADP-stimulated mitochondrial respiration underlies in vivo mitochondrial dysfunction in muscle of male type 2 diabetic patients. Diabetes 57(11): 2943-2949

[5] Hoeks J, van Herpen NA, Mensink M, et al. (2010) Prolonged fasting identifies skeletal muscle mitochondrial dysfunction as consequence rather than cause of human insulin resistance. Diabetes 59(9): 2117-2125

[6] Association AD (2019) 6. Glycemic targets: standards of medical care in diabetes—2019. Diabetes Care 42(Supplement 1): S61-S70

[7] Seaquist ER, Anderson J, Childs B, et al. (2013) Hypoglycemia and diabetes: a report of a workgroup of the American Diabetes Association and the Endocrine Society. The Journal of Clinical Endocrinology & Metabolism 98(5): 1845-1859

[8] Weir JdV (1949) New methods for calculating metabolic rate with special reference to protein metabolism. The Journal of physiology 109(1-2): 1-9

[9] Brouwer E (1957) On simple formulae for calculating the heat expenditure and the quantities of carbohydrate and fat oxidized in metabolism of men and animals, from gaseous exchange (Oxygen intake and carbonic acid output) and urine-N. Acta physiologica et pharmacologica Neerlandica 6: 795-802

[10] Steele R (1959) Influences of glucose loading and of injected insulin on hepatic glucose output. Annals of the New York Academy of Sciences 82(2): 420-430

[11] Krssak M, Brehm A, Bernroider E, et al. (2004) Alterations in postprandial hepatic glycogen metabolism in type 2 diabetes. Diabetes 53(12): 3048-3056

[12] Stadler M, Krššák M, Jankovic D, et al. (2014) Fasting and postprandial liver glycogen content in patients with type 1 diabetes mellitus after successful pancreas–kidney transplantation with systemic venous insulin delivery. Clinical endocrinology 80(2): 208-213
